# Multi-platform characterization of the human cerebrospinal fluid metabolome: a comprehensive and quantitative update

**DOI:** 10.1186/gm337

**Published:** 2012-04-30

**Authors:** Rupasri Mandal, An Chi Guo, Kruti K Chaudhary, Philip Liu, Faizath S Yallou, Edison Dong, Farid Aziat, David S Wishart

**Affiliations:** 1 Department of Biological Sciences, University of Alberta, 11455 Saskatchewan Drive, Edmonton, AB Canada T6G 2E8; 2 Department of Computing Sciences, University of Alberta, Athabasca Hall, Edmonton, AB Canada T6G 2E8; 3 National Institute for Nanotechnology, 11421 Saskatchewan Drive, Edmonton, AB, Canada T6G 2M9

## Abstract

**Background:**

Human cerebral spinal fluid (CSF) is known to be a rich source of small molecule biomarkers for neurological and neurodegenerative diseases. In 2007, we conducted a comprehensive metabolomic study and performed a detailed literature review on metabolites that could be detected (via metabolomics or other techniques) in CSF. A total of 308 detectable metabolites were identified, of which only 23% were shown to be routinely identifiable or quantifiable with the metabolomics technologies available at that time. The continuing advancement in analytical technologies along with the growing interest in CSF metabolomics has led us to re-visit the human CSF metabolome and to re-assess both its size and the level of coverage than can be achieved with today's technologies.

**Methods:**

We used five analytical platforms, including nuclear magnetic resonance (NMR), gas chromatography-mass spectrometry (GC-MS), liquid chromatography-mass spectrometry (LC-MS), direct flow injection-mass spectrometry (DFI-MS/MS) and inductively coupled plasma-mass spectrometry (ICP-MS) to perform quantitative metabolomics on multiple human CSF samples. This experimental work was complemented with an extensive literature review to acquire additional information on reported CSF compounds, their concentrations and their disease associations.

**Results:**

NMR, GC-MS and LC-MS methods allowed the identification and quantification of 70 CSF metabolites (as previously reported). DFI-MS/MS allowed the quantification of 78 metabolites (6 acylcarnitines, 13 amino acids, hexose, 42 phosphatidylcholines, 2 lyso-phosphatidylcholines and 14 sphingolipids), while ICP-MS provided quantitative results for 33 metal ions in CSF. Literature analysis led to the identification of 57 more metabolites. In total, 476 compounds have now been confirmed to exist in human CSF.

**Conclusions:**

The use of improved metabolomic and other analytical techniques has led to a 54% increase in the known size of the human CSF metabolome over the past 5 years. Commonly available metabolomic methods, when combined, can now routinely identify and quantify 36% of the 'detectable' human CSF metabolome. Our experimental works measured 78 new metabolites that, as per our knowledge, have not been reported to be present in human CSF. An updated CSF metabolome database containing the complete set of 476 human CSF compounds, their concentrations, related literature references and links to their known disease associations is freely available at the CSF metabolome database.

## Background

There is a growing need among the metabolomics and clinical communities to develop comprehensive, centralized reference resources for clinically important biofluids such as cerebrospinal fluid, blood, urine and saliva. In this regard, we have undertaken the task to systematically characterize each of these biofluids as part of the human metabolome project [1]. The first biofluid we studied in detail, in 2007, was human cerebrospinal fluid (CSF) [2]. Although CSF is not an easily accessible biofluid, its relative metabolic simplicity and potential importance to central nervous system diseases makes it particularly important in biomedical research and clinical chemistry [2]. Since the composition of CSF is directly dependent upon metabolite production rates in the brain [3], analysis of the CSF metabolome can potentially offer biochemical insights into central nervous system disorders, such as brain injury [4], Alzheimer's disease [5], Parkinson's disease [6] and multiple sclerosis [7]. Indeed, in the five years since our initial 'CSF metabolome' study was completed the CSF metabolome database [8] has been used to facilitate a wide range of metabolomic studies on central nervous system diseases, including multiple sclerosis [9], brain cancer [10] and amyotrophic lateral sclerosis [11].

At the time it was first published, the CSF metabolome database consisted of a total of 308 detectable metabolites, with extensive information on compound names, structures, identifiers, concentrations, related literature references and links to known disease associations. In that CSF study [2], we also showed that the metabolomic technologies available at that time were able to detect and quantify only about 23% of the known or detectable CSF compounds. Since that time, continuing advances in the analytical technologies for metabolomics (including improvements to instrumentation sensitivity, enhanced separation capacity, better software and more compound standards) have occurred. This technical improvement, along with the growing interest in the CSF metabolome in clinical communities has led us to re-visit the human CSF metabolome. In particular we wanted to find out if these improved technologies could lead to a substantive improvement to the level of CSF metabolite coverage achievable by standard metabolomic technologies. We also wanted to determine if new, or previously unidentified, CSF metabolites had been reported in the literature or could be discovered using these enhanced metabolomics platforms. Finally, we wanted to update the CSF metabolome database so that it contained the latest information on all known or detectable CSF metabolites, their concentrations, the latest references and their disease associations.

Here we wish to report the results of this work, including the use of five different metabolomic platforms (nuclear magnetic resonance (NMR), gas chromatography-mass spectrometry (GC-MS), liquid chromatography-mass spectrometry (LC-MS), direct flow injection-mass spectrometry (DFI-MS/MS) and inductively coupled plasma-mass spectrometry (ICP-MS)) to characterize multiple CSF samples as well as an extensive literature review (covering the period from 2008 to 2011) aimed at identifying and tabulating new (or previously unidentified) CSF metabolites along with new or updated CSF metabolite biomarkers. All of these data, along with their concentrations, related literature references and links to their known disease associations are freely available at the CSF metabolome database [8].

## Materials and methods

### Cerebral spinal fluid samples

Lumbar CSF samples were collected from patients screened for meningitis in accordance with guidelines and consent protocols established by the University of Alberta Research Ethics Board [2] and conforming to the Declaration of Helsinki principles. Only a small portion (<5%) were shown to have meningitis, suggesting that the CSF samples were from mostly neurologically normal individuals.

A more detailed list describing the patient population is shown in Table 1. The typical volume of each CSF samples was 0.5 to 1.0 ml. CSF samples were placed in a freezer for long-term storage at -80°C. All CSF samples were thawed on ice for approximately 2 h before use. A total of seven samples were used for the analyses described below.

**Table 1:** Summary of samples

Number of patients	7
Age	Adult (18 to 40 years old)
Race	White
Disease	Screened for meningitis; only a small portion (<5%) were shown to have meningitis

### NMR, GC-MS and LC-MS compound identification and quantification

Identical procedures using identical instruments, as described in [2], were employed to process and characterize metabolites from the CSF samples collected above. More specifically, all ^1^H-NMR spectra were collected on 500 μl CSF samples at 25°C (via the first transient of the tnnoesy-presaturation pulse sequence) using a 500 MHz Inova (Varian Inc., Palo Alto, CA, USA) spectrometer equipped with a 5 mm Z-gradient PFG Varian cold-probe. All ^1^H-NMR spectra were baseline corrected and analyzed using the Chenomx NMR Suite Professional software package version 6.1 (Chenomx Inc., Edmonton, AB, Canada). All GC-MS data were acquired on an HP 6890/5975 GC/MS equipped with a DB-5 column. N-methyl-N-(trimethylsilyl) trifluoroacetamide (MSTFA) derivatized extracts were prepared from 200 μl of CSF using standard methods [2]. Samples were run using full scan at a mass range of 50 to 500 m/z, with a 55 minute run time using a starting temperature of 70°C and a final temperature of 350°C. Trimethylsilated metabolites were identified using the AMDIS (automated mass spectral deconvolution and identification system) software package [12] in conjunction with the 2008 National Institute of Standards and Technology (NIST) library [13], and quantified using external multi-point calibration curves. For the LC-MS studies, CSF samples were pooled and analyzed using a Bruker Daltonics 9.4T Apex-Qe FT-ICR mass spectrometer equipped with a Waters ultra-performance liquid chromatography (UPLC) system. Spectra were collected in both positive and negative ion modes. Metabolites were identified and confirmed by high resolution Fourier transform mass spectrometry (FTMS by comparing their parent ion and fragment ion masses to known masses or fragment ion spectra from the Human Metabolome Database [14].

### DFI-MS/MS compound identification and quantification

In addition to characterizing CSF using NMR, GC-MS and LC-FTMS, we also employed a targeted quantitative metabolomics approach using direct flow injection mass spectrometry (Absolute*IDQ*™ kit). The kit is a commercially available assay from Biocrates Life Sciences AG (Innsbruck, Austria), and it was originally validated for plasma samples. Recently, the kit has been optimized for the analysis of human CSF [15] and urine samples. This kit assay, in combination with a 4000 QTrap (Applied Biosystems/MDS Sciex, Concord, Ontario, Canada) mass spectrometer, was used to identify and quantify of a large number of endogenous metabolites, including amino acids, acylcarnitines, glycerophospholipids, sphingolipids and sugars. The method combines the derivatization and extraction of analytes with the selective mass-spectrometric detection using multiple reaction monitoring (MRM) pairs. Isotope-labeled internal standards are integrated into a kit plate filter for metabolite quantification.

The Absolute*IDQ*™ kit contains a 96 deep-well plate with a filter plate attached with sealing tape, as well as reagents and solvents used to prepare the plate assay. The first eight wells in each kit are used for standardization and quality control. A straightforward sample preparation step was used for the assay, as described in the kit's user manual. CSF samples were left to thaw on ice, and then vortexed and centrifuged at 13,000 × g. A total of 30 μl of supernatant from each CSF sample was loaded on a filter paper placed on top of the kit plate and dried in a stream of nitrogen. Subsequently, 20 μl of a 5% solution of phenyl-isothiocyanate was added for derivatization. After incubation, the filter spots were dried again using an evaporator. Extraction of the metabolites was then achieved by adding 300 μl methanol containing 5 mM ammonium acetate. The extracts were obtained by centrifugation into the lower 96-deep well plate, followed by a dilution step with 600 μl of the kit's mass spectrometry running solvent. The extracts were analyzed using a 4000 QTrap (Applied Biosystems/MDS Sciex) mass spectrometer. A standard flow injection protocol consisting of two 20 μl injections (one for the positive and one for the negative ion detection mode) was applied for all measurements. MRM detection was used for quantification. Met*IQ *software, which is proprietary to Biocrates and included in the kit, was used to control the entire assay workflow. This included sample registration to automated calculation of metabolite concentrations to the export of data into other data analysis programs.

### Multi-element analysis using ICP-MS

For elemental (primarily metal) analysis by ICP-MS, seven CSF samples were processed as described previously [16]. In particular, CSF samples were sonicated in an ultrasound water bath for 10 minutes in order to obtain a homogeneous dispersion. The sample was then diluted with 2% HNO_3_. Elemental concentrations were determined on a Perkin-Elmer Sciex Elan 6000 quadrupole ICP-MS operating in a dual detector mode (Santa Clara, California, USA). Blank subtraction was applied after internal standard correction. A four point calibration curve was used to quantify compounds (0, 0.025, 0.050, and 0.100 ppm for Na; 0, 0.25, 0.50, and 1.00 ppm for Ca, Mg, Fe, K; 0, 0.005, 0.010, and 0.020 ppm for the remaining elements). The sample uptake rate was approximately 1 ml/minute with 35 sweeps per reading using one reading per replicate and three replicates. Dwell times were 10 to 20 ms for all elements with the exception of As (which was 100 ms). The relative standard deviation (2σ level) for As, Ni, Pb, and Zn was between 5 and 10%. The accuracy of the ICP-MS analytical protocol was periodically evaluated via the analysis of certified reference standard materials (whole rock powders) BE-N and DR-N available from the SARM laboratory at the CRPG (Centre de Recherches Pétrographiques et Géologiques).

### Literature survey of CSF metabolites

In addition to the experimental analysis of the CSF metabolome described above, a comprehensive literature review, covering the past 4 years (2008 to 2011) was performed to look at known or newly discovered CSF metabolites and metabolite concentrations. An in-house text-mining tool, which was originally developed for the Human Metabolome Database [14], was used to facilitate the literature survey. This program generated a hyperlinked list of abstracts and papers from PubMed containing relevant information about CSF metabolites and CSF concentration data. Keywords used for this literature search included terms such as 'CSF', 'cerebrospinal fluid', 'human', 'concentration', 'quantification' and the names of the metabolites in the Human Metabolome Database. From the resulting 106 papers and abstracts, we manually extracted metabolite information (metabolite identities, concentrations, associated disease states, and so on) and entered the data into our CSF metabolome database.

## Results and discussion

### NMR, GC-MS and LC-MS compound identification and quantification

**Figure 1:**
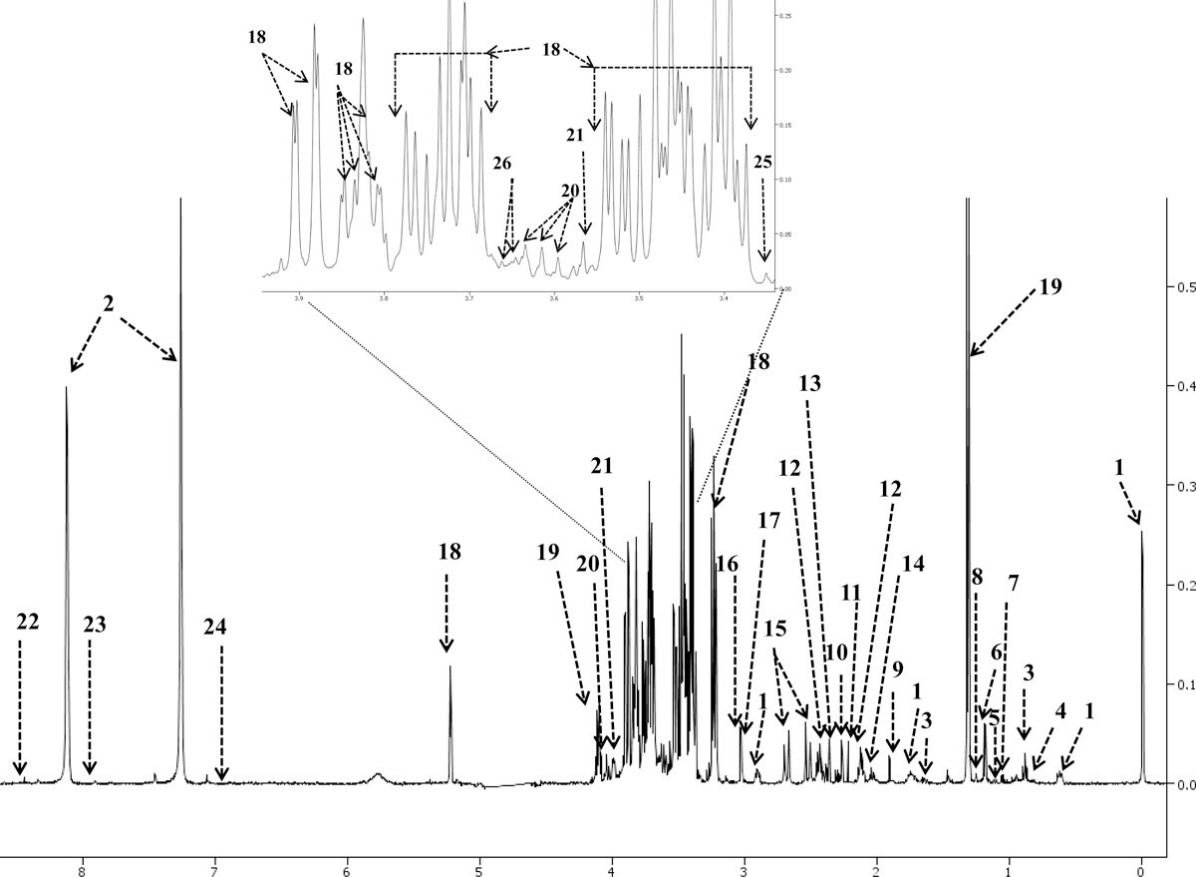
**Typical 500 MHz ^1^H-NMR spectrum of human cerebrospinal fluid**. Numbers indicate the following metabolites: 1, DSS; 2, imidazole; 3, 2-hydroxybutyric acid; 4, 2-hydroxyisovaleric acid; 5, 2-oxoisovaleric acid; 6, 3-hydroxybutyric acid; 7, 3-hydroxyisobutyric acid; 8, 3-hydroxyisovaleric acid; 9, acetic acid; 10, acetoacetic acid; 11, acetone; 12, L-glutamine; 13, pyruvic acid; 14, L-glutamic acid; 15, citric acid; 16, creatinine; 17, creatine; 18, D-glucose; 19, L-lactic acid; 20, myo-inositol; 21, D-fructose; 22, formic acid; 23, L-histidine; 24, L-tyrosine; 25, methanol; 26, glycerol.

**Figure 2:**
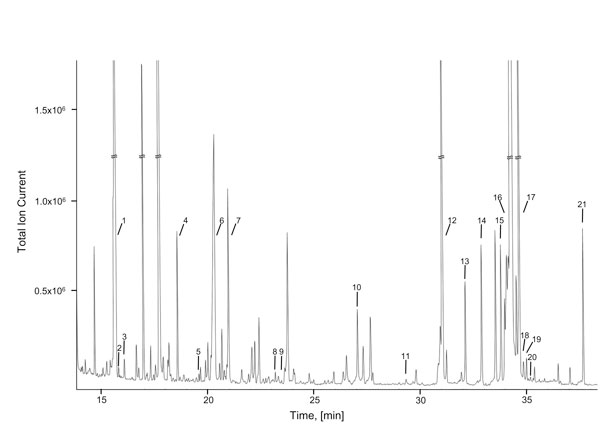
**Typical GC-MS total ion chromatogram spectrum of human cerebrospinal fluid**. Numbers indicate the following metabolites: 1, L-lactic acid; 2, oxalic acid; 3, L-alanine; 4, glycine; 5, L-valine; 6, urea; 7, glycerol; 8, L-serine; 9, L-threonine/pyroglutamic acid; 10, L-glutamine; 11, L-phenylalanine; 12, ribitol; 13, L-glutamic acid; 14, citric acid; 15, D-fructose; 16, D-glucose; 17, D-galactose; 18, L-lysine; 19, mannitol; 20, L-tyrosine; 21, myo-inositol.

A total of 53 metabolites were identified (47 quantified) by NMR, 41 metabolites were identified (15 quantified) by GC-MS, and LC-MS permitted the identification of 17 metabolites. A total of 70 non-redundant CSF metabolites were identified (62 quantified) using these three platforms. No additional metabolites beyond those originally reported in [2] were identified. No doubt the use of more modern equipment might have improved the situation, but certainly when considering NMR as a metabolomic platform, CSF spectra appear to be fully determined and fully assigned. A typical 500 MHz ^1^H-NMR spectrum and high resolution GC-MS total ion chromatogram are shown in Figures 1 and 2, respectively.

### DFI-MS/MS compound identification and quantification

**Figure 3:**
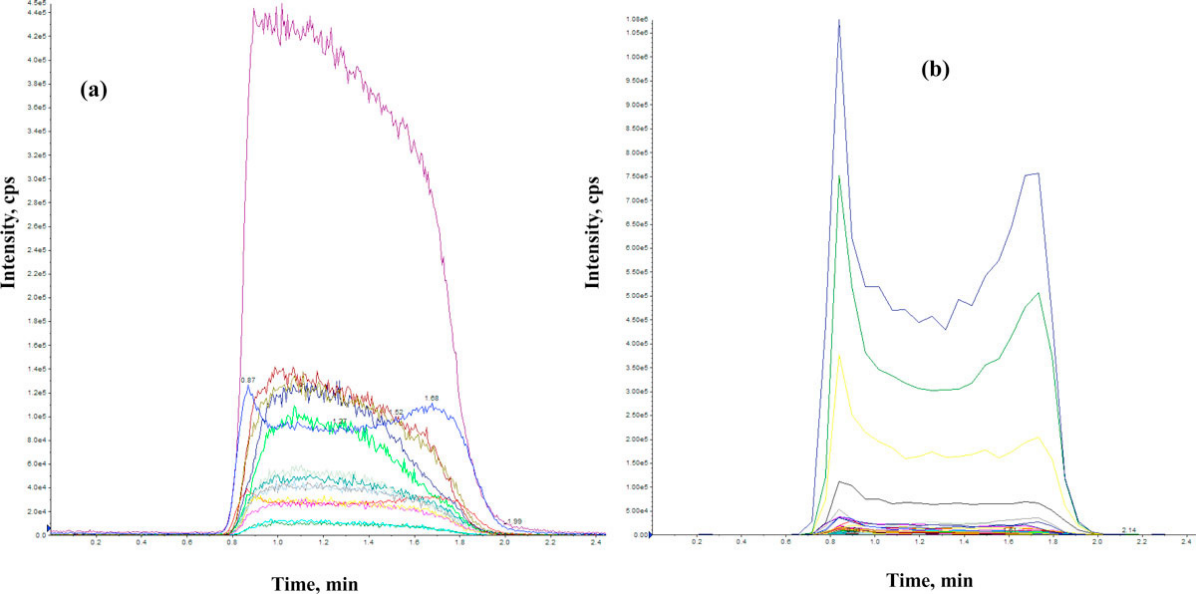
**Typical direct flow injection (DFI) spectra of human cerebrospinal fluid**. **(a) **negative mode, MRM (15 pairs); **(b) **positive mode, MRM (175 pairs).

The Biocrates Absolute*IDQ*™ kit permits the measurement of 162 metabolites (41 acylcarnitines, 14 amino acids, hexose, 76 phospatidylcholines (PCs), 15 lyso-phosphatidylcholines and 15 sphingolipids). From these 162 potentially detectable metabolites, quantitative results were obtained for 78 metabolites (6 acylcarnitines, 13 amino acids, hexose, 42 PCs, 2 lyso-phosphatidylcholines and 14 sphingolipids). These results are summarized in Table 2 and typical direct flow injection (DFI) spectra in both positive and negative modes are shown in Figure 3. The other 84 metabolites that were assayed were below the limit of detection. This result is in agreement with a previous study conducted by Biocrates (Application Note 1003-1 [15]) for pooled human CSF samples that reported a total of 65 quantified metabolites (5 acylcarnitines, 14 amino acids, hexose, 35 PCs and 10 sphingolipids).

**Table 2:** Concentrations of metabolites in human cerebrospinal fluid samples

Compound name	Average (μM)	Standard deviation (μM)	Literature value (μM)
1,5-Anhydrosorbitol^a^	25	13	18 ± 5
2-Hydroxybutyric acid^a, b^	40	24	35 ± 24
2-Hydroxyisovaleric acid^a^	8	6	7 ± 7
2-Oxoglutaric acid^a^	5	4	9 ± 3
2-Oxoisovaleric acid^a^	6	3	8 ± 7
3-Hydroxybutyric acid^a^	34	31	46 ± 24
3-Hydroxyisobutyric acid^a^	6	3	18 ± 18
3-Hydroxyisovaleric acid^a^	4	2	NA
Acetaminophen^a^	11	6	NA
Acetic acid^a^	58	27	100 ± 30
Acetoacetic acid^a^	12	14	6 ± 6
Acetone^a^	20	21	67 ± 24
L-Alanine^a^	46	27	37 ± 7
Choline^a^	3	1	8 ± 5
Citric acid^a^	225	96	176 ± 50
Creatine^a^	44	13	NA
Creatinine^a^	43	12	65 ± 25
Dimethylsulfone^a^	2	1	11 ± 6
Dimethylamine^a^	2	1	NA
Formic acid^a^	32	16	NA
D-Fructose^a^	160	91	240 ± 20
D-Glucose^a, c^	2,960	1,110	5,390 ± 1,650
L-Glutamic acid^a^	40	52	33 ± 7
L-Glutamine^a, c^	398	150	444 ± 80
Glycerol^a, b^	28	14	14 ± 3
L-Histidine^a, c^	15	8	12 ± 2
L-Isoleucine^a^	7	5	8 ± 3
Isopropyl alcohol^a^	22	56	NA
Lactic acid^a^	1,651	626	1,590 ± 330
L-Leucine^a^	16	9	19 ± 4
L-Lysine^a^	29	13	28 ± 8
D-Mannose^a^	24	13	64 ± 8
Methanol^a^	44	36	NA
L-Methionine^a, c^	5	4	6 ± 3
Myo-inositol^a^	84	40	133 ± 20
Oxalacetic acid^a^	27	15	7 ± 2
L-Phenyalanine^a, c^	15	8	18 ± 7
Propylene glycol^a^	33	50	7.3 ± 0.5
Pyroglutamic acid^a^	47	30	41 ± 30
Pyruvic acid^a^	53	42	71 ± 30
L-Serine^a, c^	38	14	42 ± 15
L-Threonine^a, c^	28	8	28 ± 5
L-Tryptophan^a, c^	10	5	2 ± 1
L-Tyrosine^a^	12	9	10 ± 4
L-Valine^a^	19	13	24 ± 7
Xanthine^a^	13	7	5 ± 1
4-Aminobutyric acid^b^	<1	<1	0.3 ± 0.1
Adenosine^b^	<1	<1	0.01 ± 0.01
Ascorbic acid^b^	178	15	164 ± 20
L-Asparagine^b^	4	2	5 ± 1
L-Aspartic acid^b^	2	1	3 ± 1
D-Galactose^b^	107	45	166 ± 99
Mannitol^b^	5	2	5 ± 1
Ribitol^b^	18	5	4 ± 12
Succinic acid^a, b^	47	12	29 ± 5
Thymine^b^	<5	<2	<1
Urea^b^	3,820	1,600	4,160 ± 1,800
Uric acid^b^	15	10	16 ± 12
DL-carnitine^c^	1.9	0.5	4.0 ± 2.0
Acetyl-L-carnitine^c^	0.322	0.148	NA
Propionyl-L-carnitine^c^	0.018	0.009	NA
Hydroxypropionylcarnitine^c^	0.063	0.030	NA
Butyryl-L-carnitine^c^	0.024	0.007	NA
Valeryl-L-carnitine^c^	0.013	0.006	NA
Glycine^b, c^	6.1	1.4	8.2 ± 3.0
L-Arginine^c^	17.9	5.7	20.2 ± 6.3
L-Ornithine^c^	4.5	2.2	6.0 ± 1.5
L-Proline^c^	1.9	1.0	4.0 ± 2.0
SM (OH) C14:1^c^	0.028	0.004	NA
SM (OH) C16:1^c^	0.020	0.007	NA
SM (OH) C22:1^c^	0.034	0.009	NA
SM (OH) C22:2^c^	0.032	0.008	NA
SM (OH) C24:1^c^	0.017	0.004	NA
SM C16:0^c^	0.336	0.109	NA
SM C16:1^c^	0.040	0.016	NA
SM C18:0^c^	0.340	0.154	NA
SM C18:1^c^	0.069	0.026	NA
SM C20:2^c^	0.003	0.001	NA
SM C24:0^c^	0.114	0.022	NA
SM C24:1^c^	0.230	0.113	NA
SM C26:0^c^	0.007	0.001	NA
SM C26:1^c^	0.007	0.003	NA
PC aa C30:0^c^	0.086	0.005	NA
PC aa C30:2^c^	0.010	0.004	NA
PC aa C32:0^c^	0.252	0.080	NA
PC aa C32:1^c^	0.150	0.051	NA
PC aa C32:2^c^	0.024	0.008	NA
PC aa C34:1^c^	1.800	0.490	NA
PC aa C34:2^c^	0.245	0.010	NA
PC aa C34:3^c^	0.038	0.003	NA
PC aa C36:1^c^	0.212	0.062	NA
PC aa C36:2^c^	0.302	0.044	NA
PC aa C36:3^c^	0.126	0.040	NA
PC aa C36:4^c^	0.228	0.036	NA
PC aa C36:5^c^	0.041	0.007	NA
PC aa C38:1^c^	0.041	0.002	NA
PC aa C38:3^c^	0.110	0.049	NA
PC aa C38:4^c^	0.209	0.068	NA
PC aa C38:5^c^	0.074	0.011	NA
PC aa C38:6^c^	0.079	0.010	NA
PC aa C40:3^c^	0.004	0.001	NA
PC aa C40:4^c^	0.015	0.007	NA
PC aa C40:5^c^	0.031	0.003	NA
PC aa C42:4^c^	0.004	0.001	NA
PC ae C32:1^c^	0.022	0.007	NA
PC ae C34:0^c^	0.018	0.005	NA
PC ae C34:1^c^	0.085	0.008	NA
PC ae C34:2^c^	0.062	0.005	NA
PC ae C34:3^c^	0.011	0.001	NA
PC ae C36:1^c^	0.032	0.007	NA
PC ae C36:2^c^	0.024	0.009	NA
PC ae C36:3^c^	0.017	0.006	NA
PC ae C36:4^c^	0.024	0.005	NA
PC ae C36:5^c^	0.032	0.005	NA
PC ae C38:1^c^	0.009	0.002	NA
PC ae C38:2^c^	0.013	0.002	NA
PC ae C38:3^c^	0.009	0.003	NA
PC ae C38:4^c^	0.020	0.005	NA
PC ae C38:5^c^	0.025	0.008	NA
PC ae C38:6^c^	0.014	0.004	NA
PC ae C40:5^c^	0.006	0.001	NA
PC ae C40:6^c^	0.018	0.003	NA
PC ae C44:4^c^	0.013	0.001	NA
PC ae C44:5^c^	0.024	0.009	NA
lysoPC a C18:0^c^	0.069	0.019	NA
lysoPC a C20:4^c^	0.024	0.007	NA

^a^Metabolites measured by NMR. ^b^Metabolites measured by GC-MS. ^c^Metabolites measured by DFI-MS/MS. aa, diacyl; ae, acyl-alkyl; NA, not available; PC, phosphatidylcholine; SM, sphingomyelin.

Of the 78 metabolites quantified by DFI-MS/MS, 11 compounds (10 amino acids and hexose/glucose) were also measurable using NMR and/or GC-MS. The measured concentrations for these common compounds on all three platforms showed very good agreement (within 15 to 30% of each other). More importantly, the DFI-MS/MS method provided quantitative data on 67 unique compounds that GC-MS, LC-MS and NMR methods could not detect. It is important to note, however, that DFI-MS/MS generates phospholipid data (PCs) that only identifies PCs by their total acyl/alkyl chain content (for example, PC aa 38:4) rather than their precise chemical structure. These 42 PC 'species' along with their concentrations are entered in our CSF metabolome database. In addition, each PC species is linked to a list of the most probable PC structures (586 in total) based on the known fatty acid compositions in humans.

### Multi-element analysis using ICP-MS

Trace metals are known to play an important role in enzyme function and a wide number of neurodegenerative diseases like Alzheimer's disease, Parkinson's disease and other related diseases [17]. A new trend in metabolomics (called 'metallomics') is evolving wherein trace metal concentrations of tissues, biofluids and even individual cells are measured. It is essential to measure trace metal concentrations in tissues and body fluids to be used as reference values. However, less data are available for normal or reference values of element concentrations for CSF samples due to the difficulties in accessing the samples and low concentrations of elements. In a recent review the importance of trace metal analysis and the potential of metal speciation analysis in CSF as a diagnostic tool for better understanding of neurodegenerative diseases were discussed [18]. An overview of current analytical techniques (like ICP-MS, inductively coupled plasma optical emission spectrometry, atomic absorption spectroscopy) and the results of total concentrations and speciation information for several elements, such as Al, As, Ca, Cd, Cu, Fe, Mg, Mn, Hg, Pb, Se and Zn, in CSF were highlighted and summarized in that review [18].

In the present paper, the metal ion composition of seven CSF samples was measured by using ICP-MS. ICP-MS is widely regarded as one of the best techniques for the characterization of the elemental composition of biological samples. This method was able to provide quantitative results for 33 metals or trace elements, as shown in Table 3. These data suggest that CSF is a reasonably rich reservoir of trace metals and that ICP-MS can be effectively used to identify and quantify a large number of trace minerals. Furthermore, our experimental results agree reasonably well with the literature values for most of the metal ions except for Al, V, Cr, Ni and As (Table 3). These concentration differences may be due to methodological or equipment differences or they may be due to regional differences with regard to environmental or occupational exposure.

**Table 3:** Multi-element analysis in cerebral spinal fluid samples using ICP-MS

Compound name	Average (μM)	Standard deviation (μM)	Literature value (μM)
Lithium (Li)	10.383	4.679	NA
Beryllium (Be)	0.045	0.019	NA
Boron (B)	3,587	1,093	NA
Sodium (Na)	109,788	10,048	145,000 ± 8,000
Magnesium (Mg)	954	207	890 ± 340
Aluminum (Al)	109	26	12.1 ± 6.3
Potassium (K)	2,163	274	2,960 ± 340
Calcium (Ca)	1047	520	1,190 ± 170
Titanium (Ti)	0.648	0.168	NA
Vanadium (V)	0.404	0.071	0.003 ± 0.001
Chromium (Cr)	0.247	0.067	0.006 ± 0.003
Iron (Fe)	9.293	1.140	5.57 ± 1.68
Manganese (Mn)	0.149	0.177	0.022 ± 0.031
Nickel (Ni)	0.097	0.109	<0.004
Copper (Cu)	2.253	3.118	1.7 ± 1.4
Zinc (Zn)	0.538	0.847	0.49 ± 0.12
Gallium (Ga)	0.011	0.003	<0.001
Arsenic (As)	0.106	0.010	0.003 ± 0.002
Selenium (Se)	0.320	0.038	0.239 ± 0.166
Rubidium (Rb)	0.499	0.097	0.620 ± 0.127
Strontium (Sr)	1.120	2.481	0.534 ± 0.079
Yttrium (Y)	0.004	0.001	NA
Zirconium (Zr)	0.277	0.086	<0.278
Molybdenum (Mo)	0.034	0.016	0.010 ± 0.009
Ruthenium (Ru)	0.005	0.002	NA
Palladium (Pd)	0.036	0.018	NA
Cesium (Cs)	0.015	0.006	0.0063 ± 0.0023
Barium (Ba)	0.223	0.236	0.130 ± 0.050
Cerium (Ce)	0.005	0.001	0.011 ± 0.005
Tantalum (Ta)	0.002	0.0009	0.0005 ± 0.0004
Platinum (Pt)	0.041	0.012	NA
Gold (Au)	0.060	0.063	0.196 ± 0.164
Lead (Pb)	0.017	0.010	0.075 ± 0.055

NA, not available.

### Literature survey of CSF metabolites

Our literature review allowed us to identify another 57 metabolites that had not been previously archived in the human CSF metabolome database. Furthermore, the literature review also allowed us to update, correct and add more than 500 new concentration ranges or averages. Additionally, dozens of new disease-metabolite associations were also identified and many older disease-metabolite associations were also updated. A total of 229 disease-metabolite associations are listed in the CSF database. In many cases, multiple concentration values are given for 'normal' conditions, in order to provide the users/readers with a better estimate of the potential concentration variations obtained by different technologies or laboratories. In general, there is good agreement between most laboratories and methods.

### The human CSF metabolome - then and now

The 2008 version of the human CSF metabolome contained 308 fully identified and quantified metabolites [2]. Of these, 70 compounds (or 23%) were shown to be routinely identifiable using a combination of NMR, GC-MS and ultra performance liquid chromatography UPLC-FTMS. Because of the very hydrophilic nature of CSF, it was shown that NMR was the most useful metabolomic platform for characterizing CSF. In 2011, thanks to an extensive literature review and additional experimental analyses, we determined that the human CSF metabolome contains at least 476 fully identified and quantified metabolites. This represents a 54% increase over the 2008 edition of the CSF metabolome. We were also able to reassess the performance of the previously used metabolomic platforms (NMR, GC-MS, UPLC-MS) as well as two more metabolomic platforms (DFI-MS/MS and ICP-MS) with regard to their efficacy in CSF metabolite characterization. Our results indicate that while essentially no improvements could be achieved using the older platforms, the addition of these two newer platforms led to significant improvements. In particular, DFI-MS/MS allowed us to identify and quantify 67 previously unmeasured compounds while ICP-MS allowed another 33 trace metals to be identified and quantified. When combined, the five platforms allowed us to measure 170 of 469 known CSF metabolites (36%). While the size of the CSF metabolome continues to grow (approximately 13% a year), the use of improved metabolomic technologies is allowing even greater coverage (growing from 23% to 36%) of the human CSF metabolome. Both trends (that is, the growth in the size of the known metabolome and the growth in metabolome coverage) are encouraging as they indicate that even though our knowledge of the CSF metabolome is rapidly expanding, our ability to characterize it is growing even faster.

## Conclusions

The main objective of this study was to advance the fields of quantitative metabolomics and global metabolic profiling to facilitate future CSF research. The updated CSF metabolome database currently contains 476 detectable metabolites. Our experimental works measured 78 new metabolites that, as per our knowledge, have not been reported to be present in human CSF. This is not a number that will remain unchanged. As technology improves, it is most likely that this number will continue to increase. However, this current set of 476 metabolites appears to provide a reasonably complete listing of the compounds that can be detected and quantified (with today's technology) in the human CSF metabolome.

## Abbreviations

CSF: cerebral spinal fluid; DFI-MS/MS: direct flow injection-mass spectrometry; FTMS: Fourier transform mass spectrometry; GC-MS: gas chromatography-mass spectrometry; ICP-MS: inductively coupled plasma-mass spectrometry; LC-MS: liquid chromatography-mass spectrometry; PC: phosphatidylcholine; MRM: multiple reaction monitoring; NMR: nuclear magnetic resonance; UPLC: ultra performance liquid chromatography.

## Competing interests

The authors declare that they have no competing interests.

## Authors' contributions

DSW conceived the project, designed the study and wrote/revised the manuscript; RM designed the experiments, contributed to experiments and data analysis, and wrote/revised the manuscript; ACG constructed the CSF metabolome database and revised the manuscript; KKC, PL, FSY, ED and FA assisted in laboratory experiments and in the CSF metabolome database construction. All authors have read and approved this manuscript for publication.
